# The Unfolded Protein Response and Diabetic Retinopathy

**DOI:** 10.1155/2014/160140

**Published:** 2014-10-29

**Authors:** Jacey Hongjie Ma, Josh J. Wang, Sarah X. Zhang

**Affiliations:** ^1^Departments of Ophthalmology and Biochemistry (Ira G. Ross Eye Institute), School of Medicine and Biomedical Sciences, University at Buffalo, The State University of New York, 308 Farber Hall, Buffalo, NY 14214, USA; ^2^SUNY Eye Institute, Buffalo, NY 14214, USA; ^3^State Key Laboratory of Ophthalmology, Zhongshan Ophthalmic Center, Sun Yat-sen University, Guangzhou 510060, China

## Abstract

Diabetic retinopathy, a common complication of diabetes, is the leading cause of blindness in adults. Diabetes chronically damages retinal blood vessels and neurons likely through multiple pathogenic pathways such as oxidative stress, inflammation, and endoplasmic reticulum (ER) stress. To relieve ER stress, the cell activates an adaptive mechanism known as the unfolded protein response (UPR). The UPR coordinates the processes of protein synthesis, protein folding, and degradation to ensure proteostasis, which is vital for cell survival and activity. Emerging evidence suggests that diabetes can activate all three UPR branches in retinal cells, among which the PERK/ATF4 pathway is the most extensively studied in the development of diabetic retinopathy. X-box binding protein 1 (XBP1) is a major transcription factor in the core UPR pathway and also regulates a variety of genes involved in cellular metabolism, redox state, autophagy, inflammation, cell survival, and vascular function. The exact function and implication of XBP1 in the pathogenesis of diabetic retinopathy remain elusive. Focusing on this less studied pathway, we summarize recent progress in studies of the UPR pertaining to diabetic changes in retinal vasculature and neurons, highlighting the perspective of XBP1 as a potential therapeutic target in diabetic retinopathy.

## 1. Introduction

Over the last two decades, diabetes mellitus (DM) has become one of the most challenging health problems worldwide. It is estimated that there were 366 million people with diabetes in 2011, and the number is expected to rise to 552 million by 2030 [1]. Potential causes for this rapid growth in the diabetic population include aging, urbanization, and increasing prevalence of obesity and physical inactivity [2]. According to The Eye Diseases Prevalence Research Group, the estimated crude prevalence rates of diabetic retinopathy (DR) and vision-threatening retinopathy are as much as 40.3% and 8.2%, respectively, in American adults 40 years and older known to have DM [3, 4]. Half of the patients with untreated proliferative retinopathy will lose their sight within five years [5]. Although good glycemic control has been shown to significantly reduce the incidence of microvascular complication, unfortunately only 17% of patients could achieve the goal of reducing glycated hemoglobin (HbA1C) to levels less than 7% [6]. Even with intensive metabolic control, up to 20% of patients will develop proliferative diabetic retinopathy (PDR) after having diabetes for 30 years [6]. In developing countries such as China and India, the prevalence of diabetes is increasing at an alarming rate [1]. Lack of a sound healthcare system and insufficient public awareness of the disease, together with uncontrolled hyperglycemia, hypertension, and hyperlipidemia, have imposed a much higher risk for the incidence of severe diabetic complications in these countries. Therefore, understanding the pathogenesis of diabetic damage, while developing novel and effective therapies to prevent diabetes-related vision loss is significant unmet needs.

In recent years, studies have demonstrated that dysfunction of the endoplasmic reticulum (ER), or ER stress, is involved in the pathogenesis of diabetes and its complications [7, 8]. The ER is the central cellular organelle responsible for protein folding and maturation. To ensure the fidelity of protein folding, the ER possesses sophisticated machinery to recognize aberrant proteins and target them for refolding or clearance [9]. This process is known as the unfolded protein response (UPR). Through comprehensive approaches that regulate ER-associated protein degradation (ERAD), protein translation, and protein folding, the UPR works toward diminishing ER stress and restoring ER homeostasis. However, when prolonged ER stress exceeds the ability of the UPR, ER homeostasis cannot be reestablished. Under these conditions, the UPR will switch from adaptive to proapoptotic actions that induce cell death to remove severely stressed and unhealthy cells (reviewed in [10]). Therefore, the central role of the UPR in human disease involves a life-or-death decision, according to an assessment of whether ER stress can be relieved in a timely fashion [11]. To date, there are three UPR branches that have been identified, namely, the inositol-requiring protein-1 (IRE1), activating transcription factor-6 (ATF6), and protein kinase RNA- (PKR-) like ER kinase (PERK) pathways. In recent years, studies have provided experimental evidence that all three UPR branches are activated in DR, contributing to retinal inflammation, apoptosis, and angiogenesis [12–18]. Among these UPR branches, the PERK pathway and its downstream target ATF4 have been thoroughly studied in retinal cells in DR, whilst the roles of the IRE1 and ATF6 pathways remain largely unexplored. X-box binding protein 1 (XBP1) is the transcription factor activated by IRE1 during ER stress. Activation of XBP1 is critical for maintaining ER function and dysregulated XBP1 expression/activity has been linked to apoptosis, cell death, and insulin resistance in diseased conditions [19]. Deletion or downregulation of XBP1 increases retinal cell sensitivity to apoptosis [20], inflammation [12], and oxidative stress [21]. However, direct evidence of XBP1 in the development of DR is still lacking. In this review, we will focus on the XBP1 pathway and provide a perspective regarding XBP1 as a potential target in DR.

## 2. Activation of the UPR Branches

**Figure 1:**
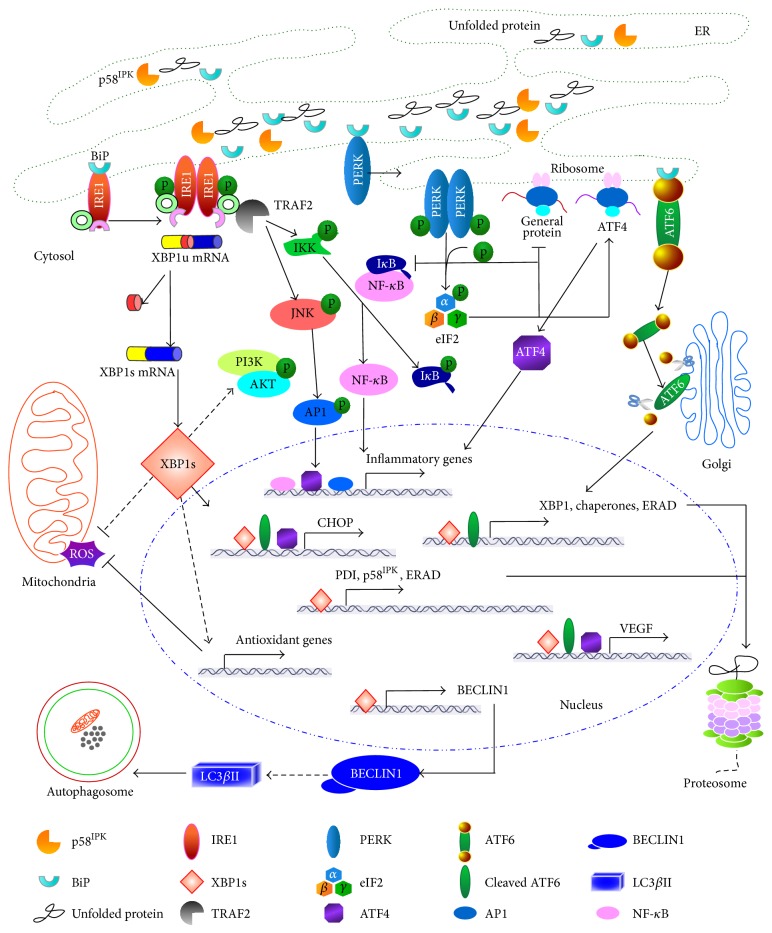
UPR signaling pathways in retinal cell physiology. Three ER stress sensors PERK, IRE1, and ATF6 are activated when unfolded proteins tether the ER chaperone BiP/GRP78 away from the sensors. Upon activation, IRE1 autophosphorylates and forms a dimer or oligomer that mediates the unconventional splicing of XBP1 mRNA. This process generates the active transcription factor XBP1s that regulates genes involved in ER-associated protein degradation (ERAD), autophagy, ER quality control, and redox homeostasis. Activated IRE1 recruits TRAF2 and induces activation of JNK and NF-*κ*B leading to increase transcription of inflammatory genes. Once removed from BiP/GRP78, AFT6 moves to the Golgi where it is cleaved by local site 1 and site 2 protease. The cleaved ATF6 is a transcription factor that regulates a subset of components for ERAD and ER chaperones. Like IRE1, PERK is activated by autophosphorylation and phosphorylates the translation initiation factor eIF2*α*, which suppresses the general translation but selectively increases the translation of ATF4. ATF4 subsequently upregulates the proapoptotic gene CHOP and also modulates the transcription of a variety of genes in inflammation, oxidative stress, apoptosis, and stress responses.

The UPR pathways are activated by three ER stress sensors located on the ER membrane, namely, IRE1, ATF6, and PERK. In resting cells, the ER luminal domain of these proteins binds to a chaperone molecule called the glucose-regulated protein 78 (GRP78, also known as immunoglobulin binding protein, BiP) and the binding prevents their activation. Upon ER stress, GRP78 is sequestered from the sensors and binds to unfolded/misfolded proteins to facilitate their refolding. The dissociation of GRP78 results in activation of the ER stress sensors and subsequently activates the UPR [19]. The three UPR pathways can be activated differentially and independent of each other and play distinct roles in inducing downstream target genes and regulating various physiological processes (Figure 1).

### 2.1. The IRE1/XBP1 Pathway

This pathway is the conserved core of UPR, because of its similarity from yeast to humans [19]. There are two different IRE1 in mammalian cells, IRE1*α* and IRE1*β*. IRE1*α* is ubiquitously expressed, while IRE1*β* is tissue specific [22, 23]. IRE1 is a bifunctional protein acting as a transmembrane kinase and endogenous ribonuclease (RNase) [24, 25]. When activated, IRE1 autophosphorylates and splices a 26-nucleotide intron from the mRNA encoding a UPR-specific transcriptional factor called XBP1, which results in a frame shift of the XBP1 gene [26]. The frame shift gives rise to spliced XBP1 or XBP1s, which is a more stable and potent activator of UPR genes [27]. XBP1s induces downstream genes, including ER chaperones and proteins involved in ERAD, and these proteins are thought to play a central role in restoring ER homeostasis and promoting cell survival [28–30].

### 2.2. The PERK/eIF2*α*/ATF4 Pathway

PERK superficially resembles IRE1, which is activated by autophosphorylation, and then phosphorylates the *α*-subunit of eukaryotic translation factor-2 (eIF2) at Ser51 with its cytoplasmic protein kinase domain [31]. This phosphorylation inhibits the reactivation of eIF2 into its GTP-bound form, resulting in global arrest of protein synthesis that, in turn, prevents further influx of ER client proteins [19, 31]. In addition to reducing translation, PERK-mediated phosphorylation of eIF2*α* also induces preferential translation of activating transcription factor 4 (ATF4), a transcription activator of the integrated stress response [32]. ATF4 induces a subset of ER stress genes involved in inflammation, apoptosis, and oxidative stress by binding to the cAMP-response element (CRE) located in their promoter or enhancer regions [33]. Two representative genes driven by ATF4 are C/EBP homologous protein (CHOP) and growth arrest and DAN-damage-inducible 34 (GADD34). CHOP is a transcription factor regulating the genes encoding components involved in apoptosis [34], while GADD34 encodes a PERK-inducible regulatory subunit of the protein phosphatase PPIC that counteracts PERK by dephosphorylating eIF2*α* [35]. Recent studies show that the PERK/p-eIF2*α*/ATF4 pathway is required not only for translational control, but also for activation of ATF6 and its target genes. The PERK pathway facilitates both the synthesis of ATF6 and trafficking of ATF6 from the ER to the Golgi for intramembrane proteolysis and activation of ATF6 [36].

### 2.3. The ATF6 Pathway

ATF6 has been identified as a basic leucine zipper (bZIP) containing transcription factor that behaves differently than the other two sensors of UPR, as it is packaged into transport vesicles and trafficked to the Golgi apparatus instead of remaining in the ER and acting via cytoplasmic effectors [37, 38]. It is then cleaved by site 1 and site 2 proteases that subsequently remove the transmembrane and luminal domains [39]. Cleaved ATF6 moves to the nucleus and binds to the promoter of ER stress response element related genes encoding ER chaperones and enzymes, such as GRP78, GRP94, and protein disulfide isomerase (PDI) [38, 40, 41]. ATF6 also regulates the transcription of other UPR targets including XBP1 [27] and CHOP [42] as well as genes involved in ER-associated degradation [19, 43, 44].

## 3. The UPR and Diabetic Retinopathy

Clinical features of DR first detected in the 19th century by ophthalmoscopy include retinal vascular abnormalities, such as microaneurysm, intraretinal hemorrhages, and cotton-wool spots [45]. With disease progression, loss of blood vessels results in retinal ischemia, which may be accompanied by venous beading, loops, and intraretinal microvascular abnormalities (IRMA). These changes eventually lead to retinal neovascularization (a hallmark of proliferative diabetic retinopathy), fibrosis, and retinal detachment. Breakdown of the blood-retinal barrier (BRB) is another important feature of DR, which can occur at any stage of the disease, advancing from macular edema and exudation in the nonproliferative phase to highly permeable neovasculature in the proliferative phase [46]. Although BRB damage primarily involves disturbed function of tight junctions between vascular endothelial cells (inner BRB) and retinal pigment epithelial cells (outer BRB), emerging evidence suggests that other neural and vascular cells (e.g., glial cells and pericytes), play a role in maintaining normal BRB function [47]. Neuronal and glial cells release metabolites, such as lactate and nitric oxide (NO), which regulate retinal blood flow to meet the metabolic needs of retinal tissue. Alterations in the interactions between neural and vascular cells contribute to vascular dysfunction in the pathogenesis of DR [45]. Although the mechanisms underlying the neurovascular dysfunction in diabetes are not fully understood, recent studies have provided some encouraging evidence suggesting a role of the UPR in DR-related retinal pathophysiology (Table 1).

### 3.1. The UPR, Inflammation, and Vascular Leakage

Recent studies have revealed that inflammation plays a causal role in diabetes-induced retinal vascular leakage. Accumulation of serum components from leaking blood vessels causes thickening of the fovea or macular edema. Diabetic retina also expresses high levels of proinflammatory cytokines, such as vascular endothelial growth factor (VEGF), tumor necrosis factor-*α* (TNF-*α*), and intercellular adhesion molecular (ICAM)-1, which increase leukocyte- (mainly monocyte/macrophage-) endothelial cell interaction and disrupt tight junctions leading to vascular leakage (reviewed in [48, 49]). Studies from several laboratories have demonstrated increased ER stress and activation of all three UPR branches in the retina in animal models of type 1 and type 2 diabetes [12–15, 18]. Increased GRP78 and ATF6 levels were also observed in retinal sections from type 2 diabetic patients with non-proliferative diabetic retinopathy [16, 17]. In contrast, local delivery of 4-phenobutyrate, a chemical chaperone that alleviates ER stress, markedly reduces retinal inflammation and attenuates vascular leakage in diabetic animals [12]. In cultured retinal endothelial cells, high glucose induces ER stress and activates the PERK-UPR downstream transcription factor ATF4. Suppressing ER stress or inhibiting ATF4 activity reduces ICAM-1, TNF-*α*, and VEGF production in high glucose-treated cells [18]. The role of ER stress and ATF4 in regulation of inflammatory factors is further confirmed in Müller cells [15], which are considered to be the major source of VEGF in diabetic retina [50]. In retinal endothelial cells, activation of XBP1 by ER stress preconditioning suppresses the TNF-*α*-induced ICAM-1 and VCAM-1 and leukocyte adhesion and ameliorates retinal vascular leakage and leukostasis. These effects appear to be mediated by an inhibition of TNF-*α*-induced NF-*κ*B activation [51]. The role of ATF6 in retinal inflammation remains to be investigated.

### 3.2. The UPR and Pericyte Loss

Pericyte loss from retinal microvessels or capillaries is an early pathological change in diabetic retina and is associated with microaneurysm formation and breakdown of the BRB. A number of studies indicate that changes in pericytes in DR can be, at least partially, attributed to ER stress. In 2006, Ikesugi and colleagues reported that reduced glucose concentration induces activation of the UPR, resulting in apoptosis in retinal pericytes [52]. In a recent study, we demonstrated that fluctuation in glucose concentrations activates the ATF/CHOP pathway of the UPR resulting in increased expression of inflammatory cytokines such as VEGF and MCP-1 in human retinal pericytes [53]. Interestingly, we found that exposure of cells to constant high glucose for 8 days does not activate the UPR in pericytes [53]; this is consistent with previous findings that glucose deprivation but not high glucose causes ER stress in pericytes [52]. These results suggest that unstable serum glucose, seen frequently in diabetic patients, is harmful to retinal pericytes and may contribute to vascular damage. Among many etiological factors, oxidative stress is a key insult responsible for vascular pathology in DR. Importantly, research by Adachi and colleagues suggests that ER stress and the UPR regulate genes involved in modulating oxidative stress, such as extracellular-superoxide dismutase (EC-SOD), an antioxidant enzyme that protects cells from oxidative injury caused by superoxide [54]. More recently, Fu and associates demonstrated that heavily oxidized and glycated low-density lipoprotein (LDL) activates oxidative stress and ER stress in human retinal pericytes, resulting in mitochondrial dysfunction, apoptosis, and autophagy [16]. These studies collectively indicate a critical role of ER stress and the UPR in pericyte loss and vascular dysfunction during DR development. However, signaling pathways of the UPR branches and reciprocal regulations between oxidative stress and ER stress, alterations in the ER and mitochondrial dysfunction, the UPR, and autophagy in diabetes-related pericyte damage remain to be investigated.

### 3.3. UPR and Angiogenesis

Retinal angiogenesis (neovascularization, NV), arising on the optic nerve and at the junction of nonperfused and perfused areas, is the hallmark of PDR, the advanced stage of DR. Untreated neovascularization leads to vitreous hemorrhage and tractional retinal detachment resulting in sudden or permanent vision loss in diabetic patients. Although the short-term effect of newly developed anti-VEGF therapy is encouraging, a large percentage of patients do not respond favorably to these therapies, suggesting there are unknown mechanisms that regulate retinal NV in diabetes (reviewed in [55]). While the UPR has long been recognized as an important mechanism for cancer cell survival, activation of the UPR by ER stress has also been shown to contribute to tumor angiogenesis [56]. As an example, the ER chaperone GRP78 plays an essential role in tumor proliferation, survival, and angiogenesis in tumor development [56]. Research from our lab demonstrated that ER stress is induced in ischemic retina and contributes to retinal NV formation in oxygen-induced retinopathy (OIR) [12]. Excessive ER stress induces VEGF in human retinal endothelial cells [57], while inhibiting ER stress by chemical chaperone 4-phenobutyrate successfully reduces VEGF expression in retinal endothelial cells and Müller cells exposed to hypoxia or high glucose and in retinas from OIR and diabetic animals [12, 15]. Furthermore, we showed that activation of ATF4 is required for hypoxia- and ER stress-induced VEGF expression [15]. More recently, a study from Ghosh and associates revealed that all three UPR branches, IRE1*α*-XBP1, PERK-ATF4, and ATF6, are involved in VEGF-A induction, which indicates an important role of the UPR in angiogenesis [58]. In contrast, Liu et al. showed that hyperglycemia or VEGF activates the IRE1*α* and ATF6 pathways but not the PERK pathway, promoting VEGF expression in retinal endothelial cells [59]. Nakamura and coworkers demonstrated that mild ER stress, induced by low-dosage tunicamycin or thapsigargin, triggers proliferation and migration of retinal endothelial cells and promotes retinal neovascularization via induction of GRP78 [60]. Future investigations are needed to decipher the exact mechanisms by which the UPR regulates endothelial angiogenic response and retinal angiogenesis.

### 3.4. The UPR and Retinal Pigment Epithelium (RPE) Dysfunction

While the majority of research on diabetes-related BRB dysfunction underlying the pathogenesis of diabetic macular edema focuses on alterations in endothelial tight junction complex and vascular leakage in DR, very few studies have looked into changes in the RPE during diabetes. Yet there is evidence from new studies as well as from studies conducted over 20 and 30 years ago suggesting that diabetes-associated damage exists in the RPE, both in animal models and in diabetic patients [61–63]. However, the effects of diabetes on the RPE barrier and other important RPE functions are poorly understood. In 2002, Abcouwer and colleagues reported that amino acid deprivation induces ER stress, resulting in VEGF upregulation in RPE cells [64]. This paper is one of the earliest studies that introduces ER stress and the UPR into retinal research and investigates a potential role of the UPR in VEGF regulation in RPE cells. In a later study, the same group demonstrated that activation of ATF4 is implicated in homocysteine-induced VEGF expression in RPE cells [65]. Furthermore, a recent study by Miranda and colleagues identified increased phosphorylation of eIF2*α*, which reflects activation of the PERK UPR branch, in RPE from diabetic patients with nonproliferative DR [66]. Similarly, Du and colleagues demonstrated that the ER chaperone GRP78 expression is increased in the RPE of diabetic subjects with retinopathy compared to those without retinopathy [17]. The role of ER stress and the UPR in diabetes-related RPE injury and potential implication in RPE-photoreceptor degeneration warrants future investigation.

### 3.5. The UPR and Retinal Neuronal Dysfunction

Eyes with DR exhibit neuronal dysfunction, including retinal ganglion cell (RGC) death, apoptosis of cells in the inner nuclear layer (INL), loss of synapses and dendrites, and related alterations in synaptic activities, as well as activation of microglial and glial cells (Müller cell and astrocytes). Growing evidence indicates that ER stress is associated with retinal neuronal cell death in several disease models [10, 67, 68]. In an experimental model of chronic glaucoma, ER stress has been shown to contribute to RGC death through activation of the PERK/p-eIF2*α*/CHOP pathway [69]. Shimazawa and colleagues reported increased ER stress in the retina correlated with apoptosis induced by N-methyl-D-aspartate (NMDA) or by raising intraocular pressure (IOP) [70]. A recent study by Oshitari and colleagues shows that enhanced PERK and CHOP levels in retinal neurons correlate with apoptosis of RGCs in retinas from diabetic rats, while similar observations were made in non-diabetic retinas after exposure to high glucose [71, 72]. Apart from a role of ER stress and the UPR in RGCs, Wu and associates demonstrated that oxidized and glycated LDL induce apoptosis of retinal Müller cells by upregulating proteins involved in UPR signaling, such as p-eIF2*α*, GRP78, CHOP, and ATF6, while activating proapoptotic Bax, caspase-3, and PARP [73]. Recently, a study by Devi and colleagues shows that exposing Müller cells to chronic hyperglycemia triggers prolonged ER stress and activates autophagy [74]. These results are consistent with our observation of increased ATF4 and CHOP activation in Müller cells, which partially colocalize with activated Müller cells in diabetic retina, suggesting that ER stress and the UPR may play a role in regulation of cellular activity of Müller cells [15].

## 4. XBP1 and Diabetic Retinopathy

**Figure 2:**
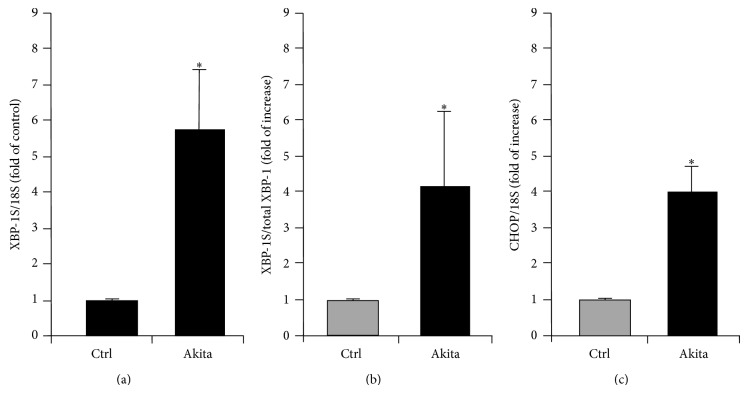
Increase in XBP1 mRNA splicing and CHOP expression in diabetic retinas. mRNA expression of spliced XBP1, total XBP1, and CHOP in retinas from 6-month-old Akita mice were determined by qRT-PCR. The values were normalized by 18S ribosome RNA and expressed as mean ± S.D. (*n* = 7 in control group and *n* = 4 in Akita group). ^*^ *P* < 0.01 versus control.

XBP1 was originally identified as a protein binding to the X-box motif in human major histocompatibility complex class II gene in early 1990s [75]. A decade later, its regulating effects on the UPR were demonstrated by two laboratories independently, as the mammalian homolog of HAC1 in yeast, which was spliced by IRE1*α* [24, 27, 76]. Over the last 10 years, the role of XBP1 splicing/activation in cell fate determination during stress has been studied extensively. XBP1 is a basic-region leucine zipper protein in the cAMP responsive element binding protein/activating transcription factor (CREB/ATF) family of transcription factors and regulates a subset of UPR target genes [30]. It is expressed ubiquitously in adult tissues as well as in fetal exocrine glands and osteoblasts [77]. XBP1 is essential for hepatocyte growth, as global knockout of XBP-1 embryos die in day 12.5 from severe liver hypoplasia and fetal anemia [78]. It is also required for ER expansion and the development of highly secretory cells such as plasma cells and pancreatic and salivary gland epithelial cells [79]. XBP1^+/−^ mice exhibit increased ER stress coupled with impaired glucose and insulin tolerance during high-fat diet (HFD)-induced obesity [7, 8]. Accumulating evidence also implicates XBP1 splicing in many disease processes such as cancer, neurodegenerative diseases, and diabetes [8, 80, 81]. Novel roles of the IRE1*α*-XBP1 branch in inflammation, cell survival, apoptosis, and angiogenesis have been revealed in recent years [21, 51, 82], and more are likely to be discovered. In diabetic Akita mice, ER stress was increased in the retina [12] accompanied by increased XBP1 splicing (Figure 2). Furthermore, preconditioning with ER stress activating XBP1 protects retinal endothelial cells from TNF-*α*-induced inflammation and retinal vascular leakage and leukostasis [51]. In the following section, we will discuss the role of XBP1 in retinal vascular and neuronal cells and its potential role in DR development as well as in future treatment strategies.

### 4.1. XBP1 and Inflammation

Accumulating evidence shows that XBP1 splicing is implicated in inflammation. Mice that lack XBP1 in intestinal epithelial cells develop spontaneous inflammation in small intestine and exhibit pathological features of human inflammatory disease (IBD). These changes are accompanied by increased ER stress and enhanced susceptibility to inflammatory stimuli [83]. In the same study, an association between XBP1 variants and both forms of human IBD was identified [83]. Mechanistically, XBP1 deficiency causes increased ER stress and IRE1*α* activation, which recruits TRAF2 and induces activation of JNK and NF-*κ*B [83]. These findings are consistent with an earlier study that demonstrates increased ER stress and JNK activation in the liver and adipose tissues, coupled with insulin resistance, in heterozygous XBP1 knockout mice challenged with a high-fat diet [8]. Likewise, heterozygous XBP1 knockout mice are more susceptible to alcohol-induced pancreatitis due to increased acinar cell apoptosis and inflammation [84]. In retinal endothelial cells, activation of XBP1 by preconditioning with ER stress attenuates TNF-*α*-stimulated NF-*κ*B activation and adhesion molecule expression [51]. Similarly, overexpressing spliced XBP1 sufficiently reduces TNF-*α*-induced IRE1*α*/IKK/NF-*κ*B activation and leukocyte-endothelial cell adhesion [51]. In these cell types, XBP1 appears to be an important modulator of inflammatory response and suppresses overt inflammation in the retina and other tissues. However, in other studies, activation of the IRE1/XBP1 UPR by mild ER stress predisposes rat pancreatic *β*-cells to an exacerbated inflammatory reaction to IL-1*β* (but not TNF-*α*) through regulation of the transcription factor fork head box O-1 (FOXO-1) and NF-*κ*B activity [85]. Activation of XBP1 also regulates production of microRNAs which in turn regulate target genes involved with immune response, such as the ER antigen peptide transporter 1 (TAP1), a key protein that coordinates the MHC class I-associated antigen presentation [86]. Discrepancies in the findings regarding the role of XBP1 in inflammation and immune response may reflect the complex and multifaceted function of XBP1 in regulation of the transcriptome.

### 4.2. XBP1 and Angiogenesis

Several studies have reported a potential role of XBP1 in the regulation of angiogenesis, yet the mechanism by which XBP1 regulates endothelial cell activity and the angiogenic process remains poorly understood. In a recent study, Liu and colleagues demonstrated that targeting the IRE1*α* or ATF6 arms of the UPR by siRNA knockdown of IRE1*α* or ATF6 significantly reduces VEGF-stimulated angiogenic response of endothelial cells and diminishes angiogenesis in mouse models of OIR or choroidal neovascularization [59]. These results are exciting in that activation of the UPR by ER stress in endothelial cells challenged with multiple potent stimulants of angiogenesis such as VEGF, hypoxia and hydrogen peroxide is clearly important for retinal angiogenesis. However, activated IRE1*α* demonstrates kinase activity that recruits and activates stress kinases such as JNK and IKK. These kinases play a proangiogenic role, independent of XBP1 splicing. Thus, future studies that separate kinase activity from endoribonuclease activity of IRE1*α* are needed to further refine the downstream pathways of IRE1 in mediating its pro-angiogenic and pro-VEGF effects.

In order to examine the role of XBP1 in endothelial cells, Zeng and associates generated endothelial cell-specific XBP1 knockout (XBP1*ecko*) mice. These mice demonstrated impaired angiogenesis in response to ischemia [87]. Furthermore, XBP1s regulated endothelial cell proliferation in a PI3K/Akt/GSK3*β*/*β*-catenin/E2F2-depandent manner [87]. In contrast, Nakamura and colleagues showed that low doses of ER stress inducers, such as tunicamycin or thapsigargin, increase the proliferation and migration of retinal endothelial cells and induce retinal neovascularization without activation of XBP1 splicing [60]. Thus, the exact role of XBP1 in angiogenesis and retinal NV is yet to be confirmed.

### 4.3. XBP1 and Autophagy

During ER stress, ERAD is activated to eliminate unfolded and misfolded protein aggregates. While misfolded polypeptides are transported to proteasome (ERAD I), large protein aggregates are primarily processed through the ER, activating autophagy (ERAD II). A low level of constitutive autophagy is also important for maintaining the quality of proteins and organelles, in order to maintain cellular function. Autophagy also serves as a crucial element of the stress response needed to protect *β*-cells under insulin-resistant states, and impaired autophagic machinery predisposes individuals to type 2 diabetes [88]. XBP1s activates transcription of major genes encoding ERAD components, such as ERdj4 [89], HRD1 [90], EDEM (ER degradation enhancing *α*-mannosidase like protein) and EDEM2 [91]. Fouillet et al. found that autophagy induced by mild ER stress selectively activates XBP1 splicing, which provides neuroprotection in Drosophila and a mouse model of Parkinson disease [92]. Interestingly, XBP1 activation in endothelial cells triggers autophagy, while knockdown of XBP1 abolishes autophagic response at the basal level or in the presence of endostatin-1 [93]. However, uncontrolled autophagy can be detrimental and cause autophagy cell death (type 2 programmed cell death). Hetz and colleagues demonstrated that downregulation of XBP1 results in uncontrolled autophagy [94, 95]. Induction of autophagy/ERAD II by the IRE1*α*/XBP1 pathway likely involves activating the ER membrane transporter AT-1 and acetylation of the autophagy protein Atg9A [94]. Thus, it appears that the IRE1*α*/XBP1 pathway plays a key role in integrating the UPR, ERAD, and autophagy to regulate cellular stress response. This notion is also supported by recent work by Adolph and colleagues demonstrating that deletion of XBP1 in intestinal epithelial cells results in ER stress, which activates PERK/eIF2*α*/ATF4 pathway and augments autophagy [96]. Loss of XBP1 also induces pIRE1-mediated NF-*κ*B activation, which is, however, restrained by autophagy. When autophagy is suppressed by deletion of autophagy-related 16-like 1 (*Atg16l1*) or autophagy-related 7 (*Atg7*) genes, a far more severe intestinal inflammation was observed in epithelial cell-XBP1-deficient mice. These results suggest a compensatory mechanism by which autophagy suppresses inflammation triggered by ER stress and XBP1 loss. Emerging evidence shows that alterations in autophagy are implicated in retinal diseases, correlating with ER stress [97–99]. In addition, hyperglycemia has been shown to activate autophagy in retinal Müller cells [74]. However, it is still not clear how ER stress-related activation of ERAD and autophagy contribute to vascular cell survival and neuronal function in diabetic retina. The role of XBP1 in ERAD and autophagy regulation in retinal cells also needs further investigation.

### 4.4. XBP1 and Retinal Cell Survival

ER stress orchestrates the process of cell death and survival depending upon the extent of UPR activation coupled with specific pathway components. It has been shown that activated XBP1 targets genes such as ER chaperones and genes responsible for ER-associated degradation (ERAD), such as P58^IPK^ and PDI-5, to aid cell survival during UPR [100]. A recent study from Ryoo and colleagues demonstrates that depletion of XBP1 in Drosophila augments photoreceptor degeneration in ninaE ^G69D^−/+, a Drosophila model for autosomal dominant retinitis pigmentosa (ADRP) suggesting that XBP1 is essential for photoreceptor survival during ER stress [20]. Our investigation demonstrated that XBP1s protects RPE and photoreceptor survival from light-induced apoptosis by suppressing oxidative stress and ER stress [21]. Consistently, mild ER stress (ER stress preconditioning) induces a selective activation of XBP1, which exerts a protective effect for cell survival [101, 102]. By contrast, sustained and full-fledged UPR involving GADD153/CHOP and ASP12/caspase 12 activation is detrimental to the cell [10]. In addition, the level and duration of XBP1 activation appears to be important for downstream signaling events and biological effects on cell fate. For example, Zeng and colleagues reported that transient activation of XBP1 splicing may increase EC proliferation, while sustained activation leads to EC apoptosis, endothelium denudation, and atherosclerotic lesion development via multiple caspase activation, as well as downregulation of VE-cadherin through gene transcriptional effects and MMP-mediated degradation [103]. Sustained expression of XBP1s induces *β*-cell dysfunction and apoptosis via inhibition of insulin, duodenal homeobox 1 (PDX1) and musculoaponeurotic fibrosarcoma oncogene homologue A (MAFA) [82].

Other gene products involved in XBP1 regulation during ER stress-induced apoptosis are the Bcl-2 family of proteins and XBP1 SUMOylation [104, 105]. SUMO is a novel ubiquitin-like protein that can covalently modify a large number of proteins and is reversed by a family of Sentrin/SUMO-specific proteases (SENPs). XBP1 can be SUMOylated during ER stress, which represses the transcriptional activity of XBP1 and leads to cell apoptosis. In recent studies, it was demonstrated that SENP1 can increase the transcriptional activity of XBP1 through regulating XBP1 SUMOylation. Loss of SENP1 enhances XBP1 SUMOylation resulting in ER stress and apoptosis [104]. Rodriguez and colleagues found that cells lacking BIM and PUMA, two novel members of Bcl-2 family of proteins, failed to maintain sustained XBP-1 splicing after prolonged ER stress that caused early inactivation of XBP1 [105]. Mutation in the BH3 domain of BIM abrogates its physical interaction with IRE1*α*, resulting in inhibition of XBP-1 splicing and reduced expression of XBP1-target genes [105]. With a Drosophila model for autosomal dominant retinitis pigmentosa, Ryoo and colleagues examined the role of XBP1 in photoreceptor cells. Their results show that reducing the expression of XBP1 accelerates photoreceptor loss and retinal degeneration [20]. In hippocampi from db/db mice, the expression of XBP1s was downregulated at both mRNA and protein levels after 24 weeks of diabetes but not in early diabetes (8 weeks), implying a potential role of XBP1 dysfunction in long-term diabetes-induced cognitive declination [81]. In contrast, overexpression of XBP1 protects RPE cells [21] and RGCs [106] from apoptosis induced by cytotoxin or axonal injury. These studies revealed the protective role of XBP1 in retinal neurons and the central nervous system. Enhancing XBP1 activity may offer a novel therapeutic strategy against retinal neuronal degeneration.

### 4.5. XBP1 and Oxidative Stress

The ER possesses a unique oxidizing environment that favors disulfide bond formation during the process of protein folding. Generation of reactive oxygen species (ROS) is a byproduct of this process. ER stress-initiated ROS generation amplifies mitochondrial ROS production and stimulates oxidative stress, which in turn enhances the ER stress response, resulting in a vicious cycle of cellular damage. Sustained ER stress and oxidative stress can lead to cell dysfunction or even cell death. Oxidative stress plays a pivotal role in the development of DR and also contributes to the resistance in reversing retinopathy after reinstitution of good glycemic control, a phenomenon known as metabolic memory [107]. Recent studies suggest UPR signaling regulates oxidative stress through transcriptional regulation of antioxidant genes. In mouse embryonic fibroblasts, deletion of XBP1 resulted in decreased expression of several antioxidant genes, including catalase, SOD1, and TRX1, augmented ROS generation and maintained P38 phosphorylation [108]. Overexpression of XBP1 restored catalase levels in XBP1-deficient cells and diminished ROS generation. Mutation analysis of the catalase promoter region revealed a potential site for direct regulation by XBP1 [108]. In RPE-specific XBP1 KO mice, the expression of antioxidant genes (SOD1, SOD2, and catalase) was significantly decreased; in contrast, the ROS level was markedly increased accompanied by enhanced oxidative damage and apoptosis of RPE cells. However, overexpression of spliced XBP1 failed to increase the expression of these antioxidant genes in RPE cells, suggesting the possibility that XBP1 indirectly regulates these genes in RPE [21]. Shao and associates reported that high glucose treatment significantly reduced XBP protein in renal mesangial cells, and simultaneously increased expression of the cytosolic NADPH oxidase subunit p47^phox^ and ROS generation [109]. Overexpression of XBP1 reversed HG-induced ROS production and reduced p47^phox^ expression, while knockdown of XBP1 led to increased ROS [109]. These findings suggest a potential role for XBP1 in maintaining cellular redox homeostasis by regulation of ROS-generating enzymes such as NADPH oxidase and antioxidant molecules; however, the exact mechanism is still not clear.

### 4.6. XBP1 and Glial Activation

Recent studies show that XBP1 splicing is involved in regulation of innate immunity [110]. Toll like receptors (TLRs) are part of the innate immune system that recognizes pathogen-associated molecules, induces inflammatory responses essential for host defenses, and initiates an adaptive immune response. TLRs are expressed in retinal cells, including RPE, microglia, Müller cells, and astrocytes [111, 112], while their role in glial activation has been studied in uveitis and age-related macular degeneration [113–115]. Retinal glial cells (Müller cells and astrocytes) and microglia are considered resident innate immune cells in the retina. These cells produce proinflammation cytokines, such as TNF-*α*, IL-1*β*, IL-8, IL-6 and ICAM in pathological conditions through activation of pathways including TLRs and a MyD88-dependent signaling cascade [113, 116]. Intravitreal injection of TLR2 agonist or Staphylococcus aureus activated TLR2 in Müller cells, resulting in the activation of NF-*κ*B and P38 MAPK in mouse retina [117]. Activation of glial cells and microglia also contributes to the pathological progression of DR, including retinal inflammation, BRB breakdown and neuronal dysfunction [118–120]. Recent studies suggest that high glucose increases oxidative stress through downregulation of thioredoxin interacting/inhibiting protein (TXNIP) in Müller cells [74]. TXNIP, also known as vitamin D3 upregulated protein (VDUP1), was first identified as an endogenous inhibitor of thioredoxin, an ROS scavenger that maintains protein cysteine sulfhydryl groups by its redox-active disulfide/dithiol sites [74]. Exposing rat Müller cells to high glucose for five days resulted in sustained upregulation of TXNIP accompanied by increased ROS generation, ATP depletion, ER stress, autophagy, inflammation and apoptosis [74]. These results indicate that a complex network of interdependent intracellular signaling pathways may contribute to diabetes-induced alternations in glial cells. In our recent study, we demonstrated that activation of ER stress by high glucose leads to increased VEGF and adhesion molecule expression through induction of ATF4 in Müller cells [15]. How other UPR branches (i.e., IRE/XBP1 and ATF6) are implicated in Müller cell activation and inflammation is currently unknown. Given the anti-inflammatory action of XBP1 in retinal endothelial cells [51], we speculate that activation of XBP1 during ER stress could reduce inflammatory cytokine production and attenuate glial cell activation in DR. Intriguingly, a recent study shows that XBP1 activation by ER stress in macrophages enhances TLR signaling and innate immune response [121]. It would be of great interest to elucidate how XBP1 regulate retinal glial cell activation and related inflammation in DR.

## 5. Conclusions and Perspectives

**Figure 3:**
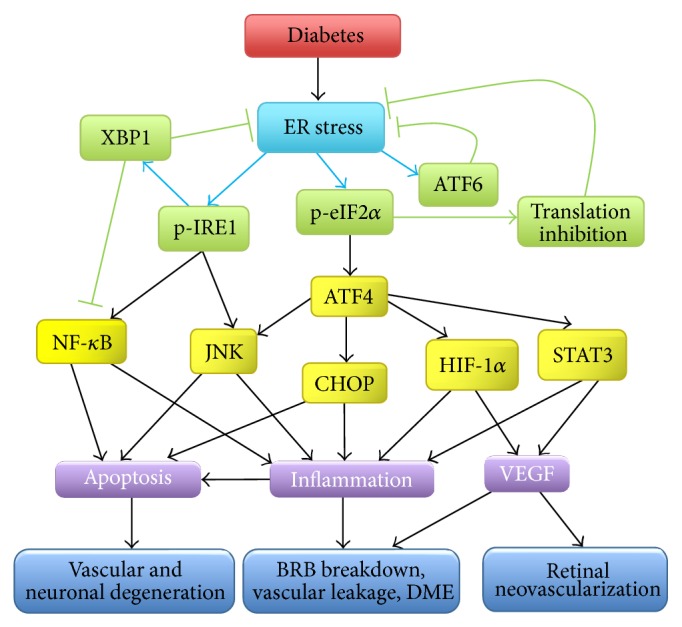
Pathogenic role of persistent ER stress in diabetic retinopathy. Chronic hyperglycemia, altered energy and metabolic hemostasis, and other detrimental factors such as oxidative stress result in ER stress in retinal cells during diabetes. While temporary and mild ER stress can be overcome by the adaptive UPR, persistent ER stress activate proinflammatory and proapoptotic signaling pathways leading to apoptosis, inflammation, increased VEGF production, and ultimately BRB breakdown, retinal NV and neuronal degeneration.

Although many clinical trials on novel therapeutic strategies have been conducted or are currently ongoing, DR remains the leading cause of blindness in working-age adults in the US. Better understanding of the cellular and molecular mechanisms underlying diabetes-related vascular and neuronal damage is important to identify therapeutic targets to protect the retina and reduce vision loss in diabetic patients. Emerging evidence suggests that increased ER stress and activation of the UPR in retinal cells promote the main pathophysiological events in DR, such as oxidative stress, inflammation, apoptosis, vascular leakage, and angiogenesis (Figure 3). Among the UPR pathways, the IRE1*α*/XBP1 branch is considered to be the core check point, and a key determinant of cell fate. Despite many elegant studies over the past decade demonstrating a crucial role of XBP1s in regulating inflammation, angiogenesis, and cell death, the exact functions of XBP1 in specific types of retinal cells and its implication in retinal adaptive response and retinal cell injury during the course of DR development and progression remain poorly understood. In addition, there is clear evidence of active cross-talk among signaling pathways of the UPR and between UPR signaling and other cellular stress responses. These complex and dynamic networks tightly control cell homeostasis and contribute to the pathogenesis of many diseases. Therefore, investigation of XBP1 in a broader range of cellular events may help us understand its role in the retina and provide new insights into the mechanisms of retinal pathology in DR.

## Figures and Tables

**Table 1:** Implications of UPR pathways in retinal pathophysiology pertinent to DR.

Branch of UPR	Cell type	Function	Involved mechanism(s)	References
IRE1/XBP1	MRPE cells, ARPE-19 cells	Apoptosis	Oxidative stress and Bcl-2 signaling	[21, 122]

IRE1/XBP1	HRECs	Hypoxia- or TNF-*α*-induced inflammation	TNF-*α*/IKK/I*κ*B/NF-*κ*B, and VEGF	[12, 51]

IRE1/XBP1	BRECs	VEGF- or high glucose-induced angiogenesis	VEGF degradation	[59]

IRE1/XBP1	ARPE19 cells	Proliferation	Not mentioned	[123]

IRE1/XBP1	ARPE19 cells	Inflammation and angiogenesis	VEGF transcriptional regulation	[124]

IRE1/XBP1	rMC-1	Inflammation and apoptosis	Oxidative stress, inflammasome, and autophagy	[74]

IRE1/XBP1	RGCs	Ischemia-, NMDA-, or IOP-induced apoptosis	Rat model, ASK1, SEK1, and pJNK	[68, 70]

ATF6	BRECs	Angiogenesis	VEGF degradation	[59]

ATF6	MIO-M1	Apoptosis	Oxidative stress	[73]

PERK/ATF4/CHOP	HRECs, TR-iBRB	Inflammation	TNF-*α*, ICAM-1, VEGF, STAT3 signaling	[12, 18]

PERK/ATF4/CHOP	rMC-1	High glucose-induced inflammation	JNK signaling, HIF-1*α*/VEGF	[15]

PERK/ATF4/CHOP	HRP	High glucose-induced inflammation	MCP-1 and VEGF	[53]

PERK/ATF4/CHOP	ARPE-19 cells	Apoptosis	Oxidative stress and mitochondrial dysfunction mediate apoptosis	[122]

PERK/ATF4	ARPE-19 cells	Inflammation and apoptosis	VEGF transcriptional regulation and oxidative stress	[65, 122, 125]

PERK/ATF4/CHOP	HRP	Apoptosis	Oxidative stress, mitochondrial dysfunction, and autophagy	[16, 52]

PERK/CHOP	hTERT-RPE	High glucose-induced apoptosis	Oxidative stress, mitochondrial dysfunction, and autophagy	[17]

PERK/CHOP	MIO-M1	Apoptosis	Oxidative stress	[73]

PERK/CHOP	RGCs, RGC-5	NMDA-, IOP-, or diabetes/high glucose-induced apoptosis	Neurotrophin-4	[69–71, 126]

HRECs: human retinal endothelial cells; HUVECs: human umbilical vein endothelial cells; HARCs: human aortic endothelial cells; BRECs: bovine retinal endothelial cells; TR-iBRB: immortalized rat retinal capillary endothelial cells; rMC-1: rat retinal Müller cells; HRP: primary human retinal pericytes; MIO-M1: human retinal Müller cells; RGCs: retinal ganglion cells; RGC-5: a rat ganglion cell line transformed with E1A virus; MRPE cells: primary mouse RPE cells; ARPE-19: an immortalized human RPE cell line; hTERT-RPE: RPE cells immortalized with hTERT.
